# Re-stratification of BI-RADS 3/4A breast lesions based on dynamic contrast-enhanced MRI: integrating cardiometabolic risk and breast microvascular remodeling phenotypes to improve diagnostic accuracy and reduce unnecessary biopsies

**DOI:** 10.3389/fphys.2026.1984062

**Published:** 2026-10-28

**Authors:** Xingzhen Hu, Fei Chen, Qianfeng Gu

**Affiliations:** 1 Department of Radiology, Ningbo Hospital of Integrated Traditional Chinese and Western Medicine, Ningbo, Zhejiang, China; 2 Women’s Health Department, Maternal and Child Health Center of Yinzhou District, Ningbo, Zhejiang, China

**Keywords:** biopsy reduction, BI-RADS 3, BI-RADS 4A, breast MRI, cardiometabolic burden, diagnostic prediction model, internal validation, perilesional vascular features

## Abstract

**Introduction:**

MRI BI-RADS 3 and 4A breast lesions occupy a narrow management boundary between imaging surveillance and biopsy. This study evaluated whether cardiometabolic context and MRI-derived perilesional vascular features improved re-stratification beyond conventional lesion-level MRI information.

**Methods:**

This retrospective diagnostic prediction-model study included 420 women with one MRI-detected lesion, comprising 160 BI-RADS 3 lesions and 260 BI-RADS 4A lesions. Malignancy was established by histopathology or, for non-biopsied lesions, by at least 24 months of imaging stability. Four prespecified ridge logistic regression models were evaluated: MRI-only, MRI plus cardiometabolic burden, MRI plus an MRI-derived vascular remodeling score, and a fully integrated model. Class weighting was not used. The complete modeling procedure, including preprocessing and penalty selection, was internally validated using 2,000 bootstrap resamples.

**Results:**

Twenty-seven lesions were malignant, including 3 BI-RADS 3 lesions and 24 BI-RADS 4A lesions. The optimism-corrected AUC was 0.760 for the MRI-only model, 0.775 for the MRI-plus-cardiometabolic model, 0.812 for the MRI-plus-vascular model, and 0.826 for the fully integrated model. The paired optimism-corrected AUC difference between the fully integrated and MRI-only models was 0.066 (95% CI 0.019–0.113; p = 0.006). The integrated model had a bootstrap-corrected calibration intercept of −0.08, calibration slope of 0.94, and Brier score of 0.052. Using aggregated out-of-bootstrap predictions, a locked risk threshold below 0.020 reassigned 91 BI-RADS 4A lesions to 6-month MRI follow-up; 90 were benign and 1 was malignant, representing a 38.1% reduction in immediate biopsy recommendations among benign BI-RADS 4A lesions. A risk threshold of at least 0.050 flagged 6 BI-RADS 3 lesions for intensified assessment, including 2 of the 3 malignant BI-RADS 3 lesions. Sensitivity, specificity, positive predictive value, and negative predictive value were 92.6%, 61.8%, 14.3%, and 99.2%, respectively.

**Discussion:**

Cardiometabolic context and MRI-derived vascular information improved internally validated risk estimation. However, the malignant downgrade and the limited number of malignant outcomes preclude clinical implementation before prospective external validation.

## 1 Introduction

Contrast-enhanced breast magnetic resonance imaging is highly sensitive for breast cancer detection and is used for screening, diagnostic problem solving, local staging, and evaluation of findings that remain uncertain after mammography or ultrasonography (Mann et al., 2019, Mann et al., 2015). Its sensitivity is accompanied by overlap in morphology and enhancement behavior between benign and malignant lesions, particularly near the lower boundary of suspicion. MRI BI-RADS 3 findings are considered probably benign and are generally managed with imaging surveillance. A systematic review reported a pooled malignancy rate of approximately 1.6%, with malignant lesions identified throughout follow-up extending to 24 months (Spick et al., 2018). BI-RADS 4A represents the adjacent low-suspicion category for which tissue diagnosis is generally recommended. The resulting management problem is asymmetric: most BI-RADS 4A biopsies are benign, but the malignant proportion remains clinically important, whereas a small number of BI-RADS 3 lesions prove malignant despite their initial surveillance assignment.

Quantitative MRI can refine categorical assessment but does not eliminate this overlap. Dynamic contrast-enhanced MRI characterizes contrast delivery, permeability, extracellular distribution, and delayed enhancement, while diffusion-weighted imaging provides information related to tissue cellularity through the apparent diffusion coefficient. In the multicenter ECOG-ACRIN A6702 study, MRI-detected malignancies had lower ADC values than benign lesions, and quantitative diffusion information reduced unnecessary MRI-prompted biopsies (Rahbar et al., 2019). A subsequent multicenter investigation showed that a predefined ADC threshold could downgrade a proportion of benign BI-RADS 4 lesions while retaining high sensitivity (Clauser et al., 2021). ADC nevertheless varies with acquisition parameters, scanner platform, lesion type, segmentation, and region-of-interest placement, and benign and malignant distributions continue to overlap (Partridge et al., 2017; Baltzer et al., 2020). Radiomics models can incorporate a broader range of imaging information, but their reported performance remains heterogeneous across acquisition, feature-extraction, and validation procedures (Lin et al., 2024). Quantitative MRI is therefore more appropriately used to modify lesion-level risk than to provide an isolated downgrade rule.

The vascular environment surrounding a lesion may supply information that is not fully represented by lesion morphology or ADC. The adjacent vessel sign has been associated with breast malignancy in histologically verified MRI cohorts, although its sensitivity is inadequate for stand-alone diagnosis (Dietzel et al., 2010; Fischer et al., 2006). DCE-MRI pharmacokinetic parameters have also been correlated with microvessel density and vascular endothelial growth factor expression (Su et al., 2003). Background parenchymal enhancement reflects contrast uptake in non-lesional fibroglandular tissue and has been associated with both breast cancer risk and MRI diagnostic performance (King et al., 2011; Thompson et al., 2019; Bechyna et al., 2025). These findings support evaluation of the lesion, its immediate perilesional region, and the surrounding parenchyma as related but non-equivalent imaging compartments. They do not establish MRI-derived vascular measurements as histologic markers of angiogenesis.

Cardiometabolic status may modify this imaging environment without functioning as an independent lesion-level cancer marker. Obesity and metabolic dysfunction have been associated with greater background parenchymal enhancement, particularly in premenopausal women (Brown et al., 2023). Blood pressure, glucose regulation, lipid abnormalities, and central adiposity may also influence endothelial function and tissue perfusion. Their diagnostic relevance is therefore conditional: cardiometabolic information may alter the interpretation of enhancement and vascular features when combined with lesion morphology, diffusion, and perilesional measurements. It should not be interpreted as evidence that metabolic syndrome or obesity independently identifies a malignant breast lesion.

Previous re-stratification studies have largely evaluated lesion morphology, enhancement kinetics, diffusion, radiomics, or individual vascular signs separately. A model that combines several domains must establish that each added block contributes information beyond conventional MRI, avoid entering both raw variables and their derived classifications into the same equation, and report performance after correction for model optimism. This requirement is especially important when malignant outcomes are uncommon. Apparent AUC estimates and reclassification results obtained from the same observations used for model development can exaggerate improvement, while class weighting can distort the predicted probabilities used to determine surveillance and biopsy thresholds.

This study therefore developed and internally validated four prespecified diagnostic models for MRI BI-RADS 3 and 4A lesions: an MRI-only model, an MRI-plus-cardiometabolic model, an MRI-plus-vascular model, and a fully integrated model. The primary objective was to determine whether the fully integrated model improved optimism-corrected discrimination, calibration, and overall probability accuracy compared with the MRI-only model. Secondary objectives were to quantify the separate incremental contribution of cardiometabolic and MRI-derived vascular information and to evaluate locked, clinically anchored probability thresholds for downgrading selected BI-RADS 4A lesions to 6-month MRI follow-up and flagging higher-risk BI-RADS 3 lesions for intensified assessment. Histopathology or at least 24 months of imaging stability served as the reference standard.

## 2 Methods

### 2.1 Study design, setting, and participants

This single-center retrospective diagnostic prediction-model study included women who underwent clinically indicated contrast-enhanced breast MRI between 5 January and 25 June 2023. The index lesion was an MRI-detected enhancing lesion assigned an original clinical BI-RADS category of 3 or 4A before biopsy, treatment, or follow-up. Outcome ascertainment ended on 30 July 2025; non-biopsied benign lesions required at least 24 months of imaging stability, and later records were not considered. The de-identified dataset was locked on 15 August 2025, and the analysis specification was locked at 09:00 on 18 August 2025 before outcome-linked model fitting. Reporting followed STARD 2015 and TRIPOD+AI guidance (Bossuyt et al., 2015; Collins et al., 2024). Ethical approval was obtained from Ningbo Hospital of Integrated Traditional Chinese and Western Medicine (Approval No. 02014068); informed consent was waived for this de-identified retrospective study.

Women aged at least 18 years with one or more MRI BI-RADS 3 or 4A lesions were eligible. One index lesion was retained per woman: the lesion with the higher category, the larger lesion when categories were identical, or the lesion listed first when diameters were equal. Exclusion criteria were an original category outside BI-RADS 3/4A; previous ipsilateral breast cancer; treatment before MRI; pregnancy or lactation; biopsy in the same quadrant within 3 months; inability to localize the lesion; duplicate records; non-diagnostic imaging; or absence of a reference standard. Image quality was graded from 0 to 4: none, mild, moderate, substantial but diagnostic, or non-diagnostic. Grades 0–3 were retained, grade 4 was excluded, and grade 3 was removed in sensitivity analysis. The cohort comprised 420 women with 420 lesions, including 160 original BI-RADS 3 and 260 original BI-RADS 4A lesions. Original category defined eligibility and baseline management; blinded adjudicated category was used for modeling. **Figure 1** presents the study pathway.

**Figure 1:**
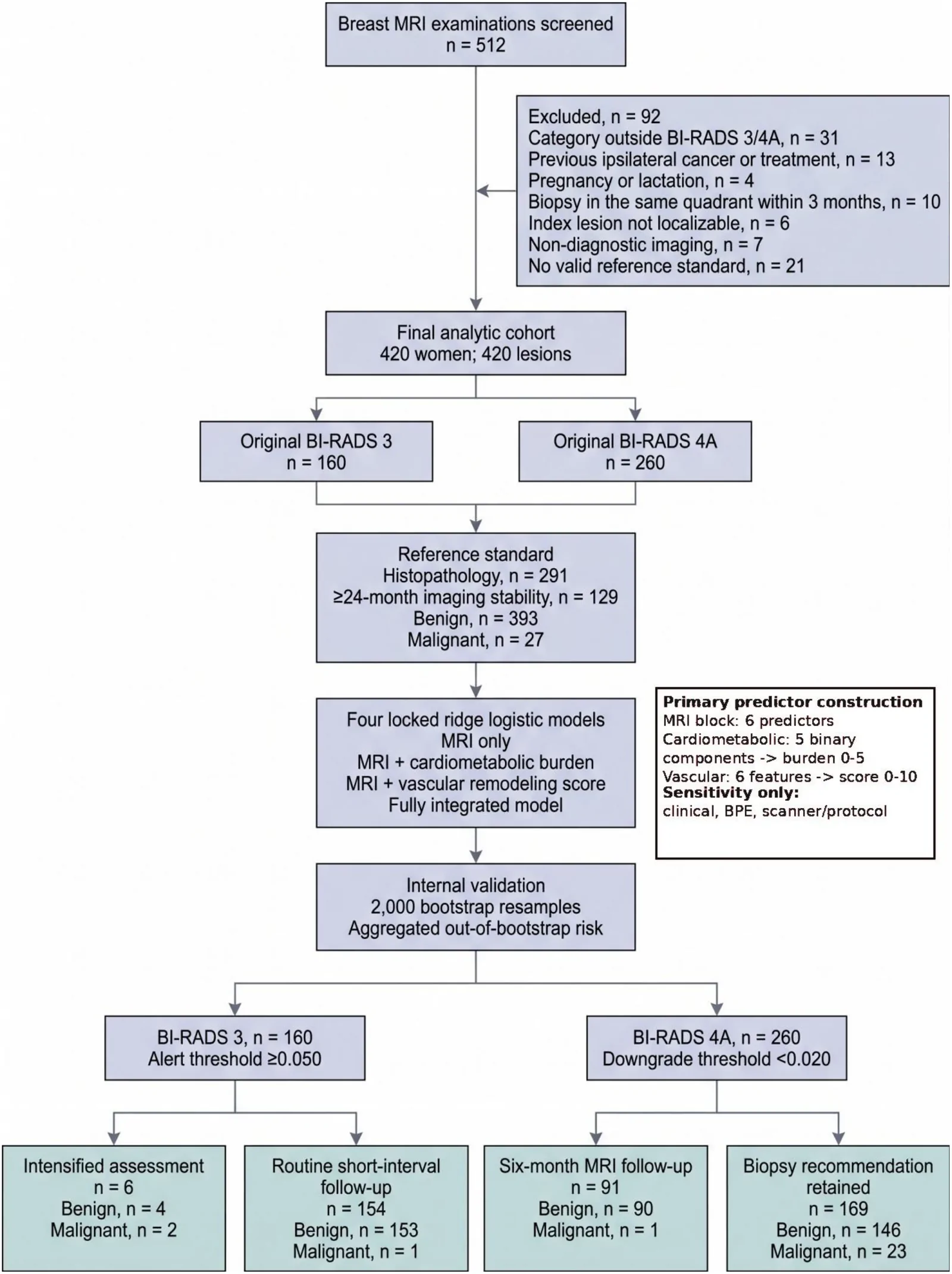
Screening, reference-standard assignment, and re-stratification pathway. Of 512 examinations screened, 420 were analyzed. Histopathology established the outcome in 291 lesions, and at least 24 months of imaging stability established benignity in 129. Four ridge logistic models were validated with 2,000 bootstrap resamples. Out-of-bootstrap risks below 0.020 identified BI-RADS 4A lesions eligible for 6-month MRI follow-up; risks of at least 0.050 flagged BI-RADS 3 lesions for intensified assessment. The added inset summarizes primary predictor construction and distinguishes variables reserved for sensitivity analysis.

### 2.2 MRI acquisition, review, and segmentation

MRI was performed prone with a dedicated bilateral coil on Siemens, GE, or Philips 1.5-T or 3.0-T systems. Examinations included fat-suppressed T2-weighted imaging, pre-contrast T1-weighted imaging, fat-suppressed three-dimensional DCE-MRI, and DWI when available. DCE-MRI contained one pre-contrast and at least 30 post-contrast phases, with 5.0–14.9 s temporal resolution and at least 360 s post-contrast acquisition. Gadolinium-based contrast material was administered at 0.1 mmol/kg and 2.0 mL/s, followed by a 20-mL saline flush. DWI used b = 0 and 800 or 1000 s/mm². Median ADC was measured in a one-voxel-eroded lesion mask and reported as ×10^-^³ mm²/s. Examinations without evaluable DWI retained a DWI-availability indicator, and missing ADC was imputed within each bootstrap development sample.

Two breast radiologists with 8 and 12 years of experience independently reviewed randomized examinations while blinded to outcomes, cardiometabolic data, and model predictions. They recorded BI-RADS category, lesion type, diameter, shape, margin, internal enhancement, initial enhancement, delayed kinetic type, background parenchymal enhancement, fibroglandular tissue amount, adjacent vessel sign, rim enhancement, and perilesional edema (D’Orsi et al., 2013). Delayed enhancement was persistent when signal increased by >10%, plateau between −10% and +10%, and washout when signal decreased by >10%. Category disagreement was resolved by blinded consensus and, when necessary, by a third radiologist with 18 years of experience. Weighted κ used the independent pre-consensus assessments.

Images were processed in 3D Slicer 5.6.2. Lesions were segmented on early subtraction images and cross-checked on pre-contrast, delayed, T2-weighted, and DWI images. Non-enhancing, hemorrhagic, necrotic, cystic, and biopsy-related regions were excluded. A 5-mm perilesional shell excluded skin, muscle, chest wall, image boundaries, and vessels >5.0 mm. Vessels were extracted with a multiscale Frangi filter using a 0.5–3.0-mm diameter range, normalized threshold of 0.18, and minimum length of 5 voxels. Vessel density was the number of centerlines intersecting the shell divided by projected shell area; asymmetry compared mirrored breasts, and tortuosity was centerline length divided by endpoint distance. **Supplementary Figure S1** shows this workflow.

Sixty-three examinations, representing 15% of the cohort, underwent blinded repeat segmentation using stratified sampling and seed 20250818. A >10% difference in lesion volume or vessel density triggered adjudication. Reproducibility used a two-way random-effects, absolute-agreement intraclass correlation coefficient. Segmentation was adequate, limited, or non-evaluable; limited quality represented 10%–25% boundary uncertainty, and >25% was non-evaluable. Non-evaluable cases were excluded, while limited cases were removed only in sensitivity analysis. **Table 1** consolidates the acquisition, assessment, and quality-control specifications.

**Table 1:** MRI acquisition, assessment, and quality-control specifications.

Domain	Specification	Analysis role
MRI system	Siemens, GE, or Philips; 1.5 T or 3.0 T	Technical description and adjustment
T2-weighted imaging	TR 3,500–6,500 ms; TE 70–110 ms; section 3.0–5.0 mm	Characterization and segmentation check
DCE-MRI	One pre-contrast and ≥30 post-contrast phases	Quantitative enhancement
DCE resolution	In-plane 0.7–1.1 mm; section 1.0–1.8 mm; 5.0–14.9 s per phase	Eligibility and technical adjustment
Contrast	0.1 mmol/kg; 2.0 mL/s; 20-mL saline flush	Protocol consistency
Post-contrast duration	≥360 s	Delayed kinetics
DWI	b = 0 and 800 or 1000 s/mm²	ADC calculation
ADC	Median in an eroded lesion mask; ×10^-^³ mm²/s	MRI predictor
DWI availability	Present or absent	Missingness indicator
Original BI-RADS	3 or 4A	Eligibility and baseline strategy
Adjudicated BI-RADS	3 or 4A	Model predictor
Morphology	Type, size, shape, margin, internal enhancement	MRI predictor block
Kinetics	Initial enhancement; persistent, plateau, or washout	MRI predictor block
Parenchyma	BPE and fibroglandular tissue amount	Descriptive and sensitivity analysis
Vascular features	Adjacent vessel, rim enhancement, edema, density, asymmetry, tortuosity	Vascular score
Lesion ROI	Three-dimensional enhancing mask	Lesion features
Perilesional ROI	5-mm shell	Perilesional features
Vessel extraction	Diameter 0.5–3.0 mm; threshold 0.18; minimum 5 voxels	Density and tortuosity
Repeat segmentation	n = 63; seed 20250818	Reproducibility
Motion artifact	Grades 0–3 retained; grade 4 excluded	Sensitivity analysis
Segmentation quality	Limited retained; non-evaluable excluded	Sensitivity analysis

ADC, apparent diffusion coefficient; BI-RADS, Breast Imaging Reporting and Data System; BPE, background parenchymal enhancement; DCE, dynamic contrast-enhanced; DWI, diffusion-weighted imaging; MRI, magnetic resonance imaging; ROI, region of interest; TE, echo time; TR, repetition time.

### 2.3 Cardiometabolic burden and adjustment variables

Cardiometabolic measurements obtained within 30 days before or after MRI were extracted from electronic records. The closest measurement was used, with the earlier value retained when dates were equally distant. Glucose and lipid measurements required at least 8 h fasting. Extracted variables were BMI, waist circumference, blood pressure, fasting glucose, HbA1c, triglycerides, HDL cholesterol, LDL cholesterol, and relevant treatment. Overweight and obesity were BMI ≥24.0 and ≥28.0 kg/m², respectively. Central obesity was waist circumference ≥85 cm (Jia et al., 2019).

Five metabolic components were coded: central obesity; blood pressure ≥130/85 mmHg or antihypertensive treatment; fasting glucose ≥5.6 mmol/L, diabetes, or glucose-lowering treatment; triglycerides ≥1.7 mmol/L or specific treatment; and HDL cholesterol <1.29 mmol/L or specific treatment. HbA1c ≥5.7% was used only in sensitivity analysis. Metabolic syndrome required at least three components (Alberti et al., 2009). Cardiometabolic burden was the component count from 0 to 5 and was the only cardiometabolic predictor in the primary models. Metabolic syndrome and descriptive burden categories—low, 0–1; moderate, 2; high, 3–5—were not entered concurrently. The component thresholds followed the prespecified definitions in **Table 2**, whereas the 0–5 cardiometabolic-burden score itself was defined *a priori* as an unweighted abnormal-component count rather than an outcome-derived score. This representation was chosen to retain one clinically interpretable predictor and to limit model dimensionality in the setting of only 27 malignant outcomes. It was not intended to imply equal biological effects of the five components. No outcome-based component weighting or separately weighted continuous cardiometabolic composite was fitted, because such alternatives would have required additional modeling assumptions and degrees of freedom in this event-limited dataset.

**Table 2:** Cardiometabolic definitions and adjustment variables.

Domain	Definition	Analysis Role
Measurement window	Closest value within ±30 days of MRI	Eligibility
Fasting requirement	≥8 h	Glucose and lipid consistency
Overweight	BMI ≥24.0 kg/m²	Description
Obesity	BMI ≥28.0 kg/m²	Description
Central obesity	Waist ≥85 cm	Burden component
Elevated blood pressure	SBP ≥130 mmHg, DBP ≥85 mmHg, or treatment	Burden component
Elevated glucose	Fasting glucose ≥5.6 mmol/L, diabetes, or treatment	Burden component
Elevated triglycerides	≥1.7 mmol/L or specific treatment	Burden component
Reduced HDL	<1.29 mmol/L or specific treatment	Burden component
HbA1c	≥5.7% sensitivity definition	Sensitivity analysis
Cardiometabolic burden	Number of abnormal components, 0–5	Primary predictor
Metabolic syndrome	≥3 components	Description
Burden category	Low, 0–1; moderate, 2; high, 3–5	Description
Menopausal status	≥12 months amenorrhea, oophorectomy, or age ≥55 years when history unavailable	Sensitivity adjustment
Hormonal exposure	Systemic hormonal therapy within 6 months	Sensitivity adjustment
Other clinical variables	Age, parity, family history, previous benign biopsy	Sensitivity adjustment
Breast composition	ACR density and fibroglandular tissue amount	Sensitivity adjustment
Technical variables	Vendor, field strength, temporal resolution, DWI availability	Sensitivity adjustment
Missing values	Bootstrap-sample median or mode	Preprocessing

ACR, American College of Radiology; BMI, body mass index; DBP, diastolic blood pressure; HDL, high-density lipoprotein; MRI, magnetic resonance imaging; SBP, systolic blood pressure.

Clinical and technical sensitivity variables were age, menopausal status, parity, family history, previous benign biopsy, hormonal exposure, ACR density, fibroglandular tissue amount, vendor, field strength, temporal resolution, and DWI availability. Postmenopausal status required ≥12 months of amenorrhea, bilateral oophorectomy, or age ≥55 years when menstrual history was unavailable. Recent hormonal exposure covered the preceding 6 months. Missing continuous and binary cardiometabolic values were imputed within each bootstrap development sample using its median or mode; burden was calculated after imputation. **Table 2** consolidates these definitions.

### 2.4 DCE-MRI, DWI, and MRI-derived vascular remodeling score

Signal intensity was measured from the three-dimensional lesion mask on the pre-contrast and all post-contrast phases. Relative enhancement at time (t) was calculated as ( $100\times [S(t)-S_{0}]/S_{0}$), where ( $S_{0}$) was pre-contrast signal intensity. Initial enhancement used the phase nearest 60 s after injection; time to peak was measured from injection to maximum relative enhancement; delayed slope was the change from peak to final-phase enhancement divided by elapsed time; signal enhancement ratio was ( $\left(S_{\text{early}}-S_{0})/(S_{\text{delayed}}-S_{0}\right)$), using phases nearest 90 s and 360 s; and iAUC60 was calculated by trapezoidal integration of relative enhancement during the first 60 s. Pharmacokinetic parameters $\left(K^{trans}),(k_{ep}),(v_{e}\right)$, and $\left(v_{p}\right)$ were estimated with the extended Tofts model and a population arterial input function (Tofts et al., 1999; Parker et al., 2006). Fits were accepted when the normalized root-mean-square error was ≤0.20 and all parameter estimates remained within predefined physiological bounds. These quantitative parameters were retained for descriptive and sensitivity analysis; iAUC60 was the only quantitative DCE variable in the primary MRI predictor block.

Median ADC was analyzed continuously, accompanied by a DWI-availability indicator; no ADC cutoff was used for re-stratification. To limit the primary model to eight effective predictor coefficients, the MRI-only block comprised adjudicated BI-RADS category, maximum lesion diameter, a binary morphologic-suspicion indicator, iAUC60, ADC, and DWI availability. Morphologic suspicion was present when a mass had an irregular shape, non-circumscribed margin, heterogeneous internal enhancement, or rim enhancement, or when non-mass enhancement had a segmental distribution, clumped enhancement, or clustered-ring enhancement. Raw morphology descriptors, kinetic type, signal enhancement ratio, and pharmacokinetic parameters were not entered simultaneously with this indicator.

The MRI-derived vascular remodeling score ranged from 0 to 10: adjacent vessel sign, 2 points; ipsilateral-to-contralateral vessel-count asymmetry ≥3, 2 points; perilesional vessel density ≥4.0 vessels/cm², 2 points; rim enhancement, 1 point; perilesional edema, 1 point; and vessel tortuosity index ≥1.25, 2 points. Background parenchymal enhancement was excluded from the score and analyzed separately. The continuous score was the sole vascular predictor in the primary models; its components and descriptive phenotypes were not entered concurrently. For descriptive reporting, scores of 0–2, 3–5, and 6–10 represented quiescent, adaptive, and remodeled phenotypes, respectively. The four models, preprocessing procedure, internal validation, and locked thresholds are summarized in **Table 3**. The component cutoffs and point weights were specified before outcome-linked model fitting as study-specific operational definitions for compressing six correlated vascular features into one predictor. They were not derived by ROC/Youden optimization or other outcome-based tuning in this cohort and were not re-estimated during model development. These exact numerical cutoffs and weights are not established BI-RADS descriptors and therefore require external validation.

**Table 3:** Predictor blocks, internal validation, and locked re-stratification rules.

Component	Variables or rule	Coding or threshold	Analysis role
Baseline strategy	Original BI-RADS 3	Short-interval follow-up	Clinical comparator
Baseline strategy	Original BI-RADS 4A	Immediate biopsy recommendation	Clinical comparator
MRI predictor 1	Adjudicated BI-RADS	3 or 4A	MRI-only model
MRI predictor 2	Maximum lesion diameter	Continuous, mm	MRI-only model
MRI predictor 3	Morphologic-suspicion indicator	Absent or present	MRI-only model
MRI predictor 4	iAUC60	Standardized continuous	MRI-only model
MRI predictor 5	ADC	Standardized continuous	MRI-only model
MRI predictor 6	DWI availability	Absent or present	MRI-only model
Cardiometabolic predictor	Abnormal metabolic-component count	Continuous, 0–5	Cardiometabolic models
Vascular predictor	MRI-derived vascular remodeling score	Continuous, 0–10	Vascular models
Model 1	MRI predictors only	Six predictor coefficients	MRI-only model
Model 2	MRI predictors plus cardiometabolic burden	Seven predictor coefficients	MRI plus cardiometabolic model
Model 3	MRI predictors plus vascular score	Seven predictor coefficients	MRI plus vascular model
Model 4	MRI predictors plus both added scores	Eight predictor coefficients	Fully integrated model
Missingness exclusion	Candidate variable missingness	>20% excluded	Predictor control
Imputation	Continuous and binary variables	Bootstrap-sample median or mode	Preprocessing
Standardization	Continuous predictors	Bootstrap-sample mean and SD	Preprocessing
Model type	Ridge logistic regression	L2 penalty; no class weighting	Probability estimation
Penalty selection	Stratified five-fold cross-validation repeated 20 times	Minimum binomial deviance	Inner model tuning
Penalty grid	100 logarithmically spaced values from ${\text{(10}}^{-4})$ to ${\text{(10}}^{2})$	Evaluated within each development sample	Tuning
Internal validation	Nonparametric bootstrap	2,000 resamples	Optimism correction
Clinical predictions	Aggregated out-of-bootstrap probabilities	One probability per lesion	Calibration and reclassification
4A downgrade rule	Integrated predicted risk	<0.020	Six-month MRI follow-up
3 alert rule	Integrated predicted risk	≥0.050	Intensified assessment
False-positive reduction	Benign 4A lesions downgraded	Count and percentage	Clinical utility
Safety outcome	Malignant 4A lesions downgraded	Count and percentage	Reclassification safety

Note. ADC, apparent diffusion coefficient; BI-RADS, Breast Imaging Reporting and Data System; DWI, diffusion-weighted imaging; iAUC60, initial area under the enhancement curve during the first 60 s; MRI, magnetic resonance imaging; SD, standard deviation.

### 2.5 Reference standard and outcome definition

The primary outcome was malignancy of the index lesion. Invasive carcinoma or ductal carcinoma *in situ* confirmed by core-needle biopsy, vacuum-assisted biopsy, surgical excision, or surgical pathology was malignant. Imaging-concordant benign pathology was benign. For non-biopsied lesions, benignity required stability, decrease, or disappearance at the index site during at least 24 months of imaging follow-up without an ipsilateral cancer diagnosis. BI-RADS 3 surveillance was scheduled at approximately 6 months (± 45 d), 12 months (± 60 d), and 24 months (± 90 d) (D’Orsi et al., 2013; Panigrahi et al., 2019). For this study, stability required <20% increase in maximum diameter and no new suspicious morphology or category upgrade. An increase of ≥20%, a new suspicious correlate, or category progression prompted tissue diagnosis.

BI-RADS 4A lesions required tissue diagnosis unless biopsy was declined after multidisciplinary review, in which case at least 24 months of imaging stability was required. Imaging-pathology concordance was assessed within 14 d of pathology availability. Discordant benign findings required repeat biopsy or surgical excision within 30 d, unless multidisciplinary review documented imaging surveillance. Reference outcomes were assigned before model predictions were calculated and without access to cardiometabolic or vascular scores.

The baseline strategy assigned all original BI-RADS 3 lesions to short-interval follow-up and all original BI-RADS 4A lesions to biopsy. Under the integrated strategy, BI-RADS 4A lesions with risk <0.020 were reassigned to 6-month MRI follow-up, whereas BI-RADS 3 lesions with risk ≥0.050 underwent intensified assessment. Intensified assessment comprised targeted ultrasound, blinded radiology re-review, earlier MRI, or biopsy when a concordant suspicious correlate was present.

### 2.6 Sample size, missing data, and bias control

The cohort size was fixed by all eligible examinations within the study period. Design adequacy was evaluated before model fitting using an assumed malignancy prevalence of 6.0%, anticipated Cox-Snell (R^2) of 0.18, maximum of eight effective predictor coefficients, and target global shrinkage of at least 0.90. The resulting minimum was 417 lesions with at least 25 malignant outcomes; the available cohort of 420 lesions and 27 malignancies met this requirement (Riley et al., 2020). The limited event count was addressed by restricting predictor burden, using ridge penalization without class weighting, and validating the entire preprocessing and fitting procedure by bootstrap resampling.

Duplicate records were identified by study identifier, MRI accession number, examination date, and laterality. Variables with >20% missingness were excluded from primary modeling. Within each bootstrap sample, missing continuous values were replaced by the development-sample median and missing binary values by its mode; standardization parameters were also estimated only from the development sample. These parameters were then applied unchanged to the corresponding validation observations. True biological outliers were retained. Values outside physiological or acquisition ranges were verified against source records and set to missing when correction was impossible. The final model was fitted to the complete cohort using preprocessing parameters estimated from the full dataset, whereas all reported internal-validation performance was based on bootstrap-corrected or aggregated out-of-bootstrap estimates.

Readers were blinded to reference outcomes and cardiometabolic data, and reference outcomes were assigned independently of model predictions. Thresholds were locked before validation and were not re-estimated after performance review. Sensitivity analysis excluded grade-3 motion artifact, limited segmentation, and examinations without evaluable DWI; restricted analysis to histopathologically verified lesions; added technical or clinical adjustment variables; added background parenchymal enhancement to the integrated model; applied the HbA1c-based glucose definition; and varied both clinical thresholds. The variable dictionary and sensitivity rules are presented in **Supplementary Table S1**.

### 2.7 Model development and locked re-stratification rule

Four prespecified diagnostic models were evaluated to separate the incremental contributions of cardiometabolic and vascular information. Model 1, the MRI-only model, included adjudicated MRI BI-RADS category, maximum lesion diameter, the prespecified morphologic-suspicion indicator, iAUC60, ADC, and DWI availability. Model 2 added the cardiometabolic component count to Model 1. Model 3 added the microvascular remodeling score to Model 1. Model 4, the fully integrated model, included the six MRI predictors, cardiometabolic component count, and microvascular remodeling score, corresponding to eight effective predictor coefficients. Cardiometabolic risk category and microvascular phenotype were retained for descriptive analysis but were not entered with their corresponding continuous scores because of redundancy. No stepwise selection, data-driven predictor screening, or interaction testing was performed. Predictor definitions, preprocessing procedures, validation methods, and re-stratification thresholds are summarized in **Table 3**. Predictor construction followed a three-block hierarchy: six MRI predictors formed the baseline block; five dichotomized metabolic components were summed into one cardiometabolic-burden predictor; and six vascular features were summarized in one vascular-remodeling score. Clinical, breast-composition, and scanner/protocol variables were reserved for sensitivity analysis and were not part of the primary eight-coefficient integrated model.

All models were fitted using L2-penalized logistic regression without outcome-based class weighting. Missing continuous variables were replaced by the median and missing categorical variables by the mode estimated within each model-development sample. Continuous predictors were then centered and scaled within the same sample, and binary predictors were coded as 0 or 1. The ridge-penalty parameter was selected independently within each development sample using stratified five-fold cross-validation repeated 20 times over 100 logarithmically spaced values from $\left(10^{-4}\right)$ to $\left(10^{2}\right)$.

Because only 27 malignant outcomes were available, the cohort was not divided into separate training and test sets. Internal validation used 2,000 bootstrap resamples, each containing 420 observations sampled with replacement. Imputation, standardization, penalty selection, and model fitting were repeated in every resample to incorporate uncertainty from the complete modeling process. Each fitted model was evaluated in its bootstrap sample and in the original cohort, and the mean performance difference was used to estimate optimism and obtain optimism-corrected discrimination and calibration measures (Harrell et al., 1996). For clinical reclassification, predictions obtained for each lesion when that lesion was not included in a bootstrap resample were averaged to obtain an aggregated out-of-bootstrap predicted probability. Final model coefficients were estimated from the complete cohort after internal validation.

The management thresholds were specified before validation and were not optimized against the observed classification results. An initial BI-RADS 4A lesion was eligible for reclassification to 6-month MRI follow-up when its aggregated out-of-bootstrap probability of malignancy was below 0.020. An initial BI-RADS 3 lesion was flagged for intensified assessment when its probability was at least 0.050. Intensified assessment consisted of targeted ultrasound, focused radiologic review, earlier MRI surveillance, or biopsy when a concordant suspicious finding was identified; it did not constitute an automatic biopsy recommendation. For classification analysis, BI-RADS 4A lesions retained for biopsy and BI-RADS 3 lesions receiving a high-risk alert were classified as test-positive, whereas downgraded BI-RADS 4A lesions and BI-RADS 3 lesions remaining under routine surveillance were classified as test-negative. The 0.020 downgrade threshold was clinically anchored to the conventional upper malignancy-risk boundary for a probably benign BI-RADS 3 assessment (D’Orsi et al., 2013). The 0.050 threshold was selected *a priori* as a pragmatic alert threshold above that boundary to identify a small subset of BI-RADS 3 lesions with materially elevated predicted risk for intensified assessment; it was not intended as a BI-RADS category cutoff or an automatic biopsy threshold. Neither threshold was optimized against the observed outcomes, and both should be regarded as prespecified working management thresholds pending external validation.

### 2.8 Statistical analysis

Continuous variables were reported as mean with standard deviation when the absolute skewness was no greater than 1.0 and as median with interquartile range otherwise. Categorical variables were reported as counts and percentages. Malignant and benign groups were compared using the Welch t test for approximately symmetric continuous variables, the Mann–Whitney U test for skewed variables, and the Pearson χ² test for categorical variables when all expected cell counts were at least 5. Fisher’s exact test was used when any expected cell count was below 5. These comparisons were descriptive, were not used for predictor selection, and were not adjusted for multiplicity.

Inter-reader agreement for MRI BI-RADS category was evaluated using weighted κ with a 95% confidence interval. Reproducibility of lesion volume, perilesional vessel density, vessel-count asymmetry, and vessel tortuosity in the repeated-segmentation subset was evaluated using a two-way random-effects, absolute-agreement, single-measure intraclass correlation coefficient with a 95% confidence interval.

Model discrimination was quantified using the area under the receiver operating characteristic curve. Overall accuracy and calibration were assessed using the Brier score, Brier skill score relative to a prevalence-only model, calibration intercept, calibration slope, and graphical agreement between predicted and observed risks. Reported primary performance estimates were corrected for optimism through bootstrap validation. Differences in AUC between models were calculated within paired bootstrap resamples so that every comparison incorporated the same resampling, preprocessing, penalty selection, and model-fitting procedures. The prespecified primary comparison was the fully integrated model versus the MRI-only model. Secondary comparisons comprised Model 2 versus Model 1, Model 3 versus Model 1, Model 4 versus Model 2, and Model 4 versus Model 3; their p values were adjusted using the Holm method.

Sensitivity, specificity, positive predictive value, and negative predictive value were calculated from the locked re-stratification rule using aggregated out-of-bootstrap probabilities. Corresponding 95% confidence intervals were calculated with the Wilson method. Decision-curve analysis compared each model with biopsy-all and biopsy-none strategies across malignancy-risk thresholds from 0.01 to 0.10, representing the clinical range in which management may shift between imaging surveillance and tissue diagnosis (Vickers and Elkin, 2006).

False-positive biopsy reduction was defined as the number of benign initial BI-RADS 4A lesions reassigned from immediate biopsy to 6-month MRI follow-up, divided by the total number of benign BI-RADS 4A lesions. The safety endpoint was the number and proportion of malignant BI-RADS 4A lesions assigned to follow-up. For BI-RADS 3 lesions, the alert yield was reported as the number of malignant lesions among those flagged for intensified assessment, together with the proportion of all malignant BI-RADS 3 lesions identified.

Data management and descriptive verification were performed using IBM SPSS Statistics 30.0.0. Model fitting, bootstrap validation, calibration, paired model comparisons, and figure generation were performed in R 4.5.1 using glmnet 4.1-8, pROC 1.18.5, mice 3.18.0, rmda 1.6, and ggplot2 3.5.2. Key model predictions and performance estimates were independently reproduced in Python 3.12.5 using pandas 2.2.2, NumPy 2.0.1, scikit-learn 1.5.2, and statsmodels 0.14.2. The random seed was fixed at 20250818 before resampling and model fitting. All tests were two-sided, and (p<0.05) was considered statistically significant for the prespecified primary comparison. Adjusted secondary model comparisons and sensitivity analysis were interpreted as supportive evidence.

## 3 Results

### 3.1 Study cohort and reference-standard outcomes

Of 512 breast MRI examinations screened, 92 were excluded because the initial MRI category was outside BI-RADS 3 or 4A (n = 31), previous ipsilateral breast cancer or treatment was present (n = 13), pregnancy or lactation was documented (n = 4), biopsy had been performed in the same quadrant within the preceding 3 months (n = 10), the index lesion could not be reliably localized (n = 6), image-quality grade 4 prevented diagnostic assessment (n = 7), or the required reference standard was unavailable (n = 21). The final cohort comprised 420 women with 420 index lesions: 160 initial BI-RADS 3 lesions and 260 initial BI-RADS 4A lesions. Participant selection and reference-standard assignment are shown in **Figure 1**.

Twenty-seven lesions were malignant, giving an overall prevalence of 6.4%. Malignancy was identified in 3 of 160 initial BI-RADS 3 lesions (1.9%) and 24 of 260 initial BI-RADS 4A lesions (9.2%; p = 0.003). Histopathologic verification was available for 291 lesions (69.3%); the remaining 129 lesions were classified as benign after at least 24 months of imaging stability. All 260 initial BI-RADS 4A lesions underwent tissue diagnosis. Among the BI-RADS 3 lesions, 129 completed imaging surveillance, 18 underwent patient-preference biopsy, 10 underwent targeted ultrasound-guided biopsy, 2 underwent biopsy after imaging upgrading, and 1 underwent biopsy after interval progression. After blinded reader review and adjudication, 159 lesions were classified as BI-RADS 3 and 261 as BI-RADS 4A. Cohort characteristics and reference-standard distributions are presented in **Table 4**.

**Table 4:** Cohort characteristics according to final reference outcome.

Variable	Benign *n*=393	Malignant *n*=27	P value
Age, years	47.23 ± 9.92	49.73 ± 10.34	0.233
BMI, kg/m²	23.60 ± 3.18	23.14 ± 2.80	0.42
Waist circumference, cm	82.54 ± 6.34	80.74 ± 5.39	0.107
Postmenopausal	155 (39.4%)	15 (55.6%)	0.099
First-degree family history	51 (13.0%)	5 (18.5%)	0.384
Recent hormonal exposure	71 (18.1%)	2 (7.4%)	0.196
ACR density A/B/C/D	11/114/175/93	1/6/12/8	0.838
Initial BI-RADS 3	157 (40.0%)	3 (11.1%)	0.003
Initial BI-RADS 4A	236 (60.0%)	24 (88.9%)	
Metabolic syndrome	73 (18.6%)	10 (37.0%)	0.02
Metabolic-component count, 0–5	1.58 ± 1.09	2.00 ± 1.39	0.137
Cardiometabolic risk: low	197 (50.1%)	12 (44.4%)	0.053
Cardiometabolic risk: moderate	123 (31.3%)	5 (18.5%)	
Cardiometabolic risk: high	73 (18.6%)	10 (37.0%)	
Maximum lesion diameter, mm	11.94 ± 5.01	14.94 ± 3.88	<0.001
Morphologic-suspicion indicator present	126 (32.1%)	20 (74.1%)	<0.001
iAUC60	20.65 ± 5.47	25.27 ± 4.18	<0.001
ADC, ×10^-^³ mm²/s	1.227 ± 0.216	1.073 ± 0.203	<0.001
DWI available	354 (90.1%)	24 (88.9%)	0.743
Adjacent vessel sign	130 (33.1%)	16 (59.3%)	0.006
Vessel-count asymmetry ≥3	104 (26.5%)	14 (51.9%)	0.005
Perilesional vessel density, vessels/cm²	3.43 ± 1.04	3.73 ± 0.79	0.071
Rim enhancement	35 (8.9%)	8 (29.6%)	0.003
Perilesional edema	28 (7.1%)	6 (22.2%)	0.015
Vessel tortuosity index	1.185 ± 0.112	1.266 ± 0.105	<0.001
Microvascular remodeling score, 0–10	3.05 ± 1.93	4.56 ± 1.80	<0.001
Quiescent phenotype	172 (43.8%)	4 (14.8%)	<0.001
Adaptive phenotype	171 (43.5%)	12 (44.4%)	
Remodeled phenotype	50 (12.7%)	11 (40.7%)	
Moderate or marked BPE	14 (3.6%)	2 (7.4%)	0.275

Reference-standard distribution			
Reference standard	Benign	Malignant	Total
Core-needle biopsy	173	19	192
Vacuum-assisted biopsy	53	4	57
Surgical pathology	10	1	11
Targeted ultrasound-guided biopsy	10	0	10
Patient-preference biopsy	18	0	18
Biopsy after MRI upgrading	0	2	2
Biopsy after interval progression	0	1	1
≥24-month imaging follow-up	129	0	129

Values are mean ± SD or n (%). ADC summaries were based on examinations with evaluable DWI. P values represent unadjusted outcome-group comparisons and were not used for predictor selection. ACR, American College of Radiology; ADC, apparent diffusion coefficient; BI-RADS, Breast Imaging Reporting and Data System; BMI, body mass index; BPE, background parenchymal enhancement; DWI, diffusion-weighted imaging. No multiplicity adjustment was applied to these descriptive comparisons; individual p values should therefore not be interpreted as confirmatory evidence.

### 3.2 Reader agreement, segmentation reproducibility, and data completeness

The two primary readers assigned the same BI-RADS category in 393 of 420 examinations (93.6%). Weighted κ was 0.865, with a 95% confidence interval of 0.813–0.917. Of the 27 disagreements, 23 were resolved by consensus and 4 required third-reader adjudication. In the repeated-segmentation subset of 63 examinations, the intraclass correlation coefficient was 0.94 (95% CI 0.90–0.96) for lesion volume, 0.90 (95% CI 0.84–0.94) for perilesional vessel density, 0.88 (95% CI 0.81–0.92) for vessel-count asymmetry, and 0.87 (95% CI 0.79–0.92) for vessel tortuosity. Six repeated segmentations exceeded the prespecified 10% disagreement threshold and underwent joint adjudication.

No retained variable exceeded the 20% missingness threshold. DWI was unavailable in 42 examinations (10.0%), all of which were prospectively identified by the DWI-availability indicator. Among the remaining measurements, missing values occurred most frequently for lesion (16/420, 3.8%), followed by HbA1c (11/420, 2.6%), waist circumference (10/420, 2.4%), triglycerides (8/420, 1.9%), HDL cholesterol and perilesional (7/420 each, 1.7%), and lesion and perilesional (6/420 each, 1.4%). Twenty examinations had grade-3 motion artifact and 19 had limited segmentation quality. Missingness patterns and quality-control classifications are reported in **Supplementary Table S1**.

### 3.3 Diagnostic model performance and internal validation

The four prespecified models were fitted to the complete cohort and internally validated through 2,000 bootstrap resamples. Apparent AUC increased from 0.783 for the MRI-only model to 0.853 for the fully integrated model. After correction for optimism, the AUCs were 0.760 for the MRI-only model, 0.775 for the MRI-plus-cardiometabolic model, 0.812 for the MRI-plus-vascular model, and 0.826 for the fully integrated model. The prespecified paired comparison showed an optimism-corrected AUC difference of 0.066 (95% CI 0.019–0.113; p = 0.006) between the fully integrated and MRI-only models.

The cardiometabolic block alone produced a small increase over the MRI-only model (ΔAUC = 0.015, 95% CI −0.006 to 0.036; Holm-adjusted p = 0.244). The vascular score produced a larger increase (ΔAUC = 0.052, 95% CI 0.013–0.091; Holm-adjusted p = 0.018). The fully integrated model also exceeded the MRI-plus-cardiometabolic model (ΔAUC = 0.051, 95% CI 0.012–0.090; Holm-adjusted p = 0.018), whereas its difference from the MRI-plus-vascular model was smaller and uncertain (ΔAUC = 0.014, 95% CI −0.006 to 0.034; Holm-adjusted p = 0.247). The smaller cardiometabolic increment and its confidence interval crossing zero indicate that the added value of this block was imprecisely estimated.

Aggregated out-of-bootstrap predictions showed progressive improvement in overall prediction error and calibration across the four models. The fully integrated model had a Brier score of 0.052, a calibration intercept of −0.08, and a calibration slope of 0.94. The corresponding MRI-only estimates were 0.057, −0.14, and 0.87. Complete discrimination and calibration estimates are presented in **Table 5**. Calibration slopes remained below 1.0, including 0.94 for the fully integrated model, consistent with residual overfitting despite optimism correction.

**Table 5:** Discrimination and calibration of the four diagnostic models.

Performance measure	MRI only	MRI + cardiometabolic burden		MRI + vascular score		Fully integrated
Apparent AUC	0.783	0.799		0.838		0.853
Optimism-corrected AUC	0.760	0.775		0.812		0.826
95% CI for corrected AUC	0.670–0.838	0.687–0.850		0.733–0.875		0.750–0.887
Out-of-bootstrap Brier score	0.057	0.056		0.054		0.052
Brier skill score	0.053	0.070		0.103		0.136
Calibration intercept	−0.14	−0.12		−0.09		−0.08
Calibration slope	0.87	0.89		0.92		0.94

Paired incremental comparisons						
Comparison		ΔAUC	95% CI		P value	
Fully integrated vs MRI only		0.066	0.019–0.113		0.006	
MRI + cardiometabolic burden vs MRI only		0.015	−0.006 to 0.036		0.244	
MRI + vascular score vs MRI only		0.052	0.013–0.091		0.018	
Fully integrated vs MRI + cardiometabolic burden		0.051	0.012–0.090		0.018	
Fully integrated vs MRI + vascular score		0.014	−0.006 to 0.034		0.247	

AUC estimates were corrected for optimism using 2,000 bootstrap resamples. Brier scores, Brier skill scores, and calibration estimates were calculated from aggregated out-of-bootstrap predictions. The fully integrated versus MRI-only comparison was prespecified as primary. Secondary-comparison p values were adjusted using the Holm method.

The internally validated ROC and calibration curves are shown in **Figures 2A**, **B**, respectively. The fully integrated model provided greater net benefit than the MRI-only model across threshold probabilities from 0.02 to 0.08; at a threshold of 0.05, net benefit was 0.026 for the integrated model and 0.018 for the MRI-only model.

**Figure 2:**
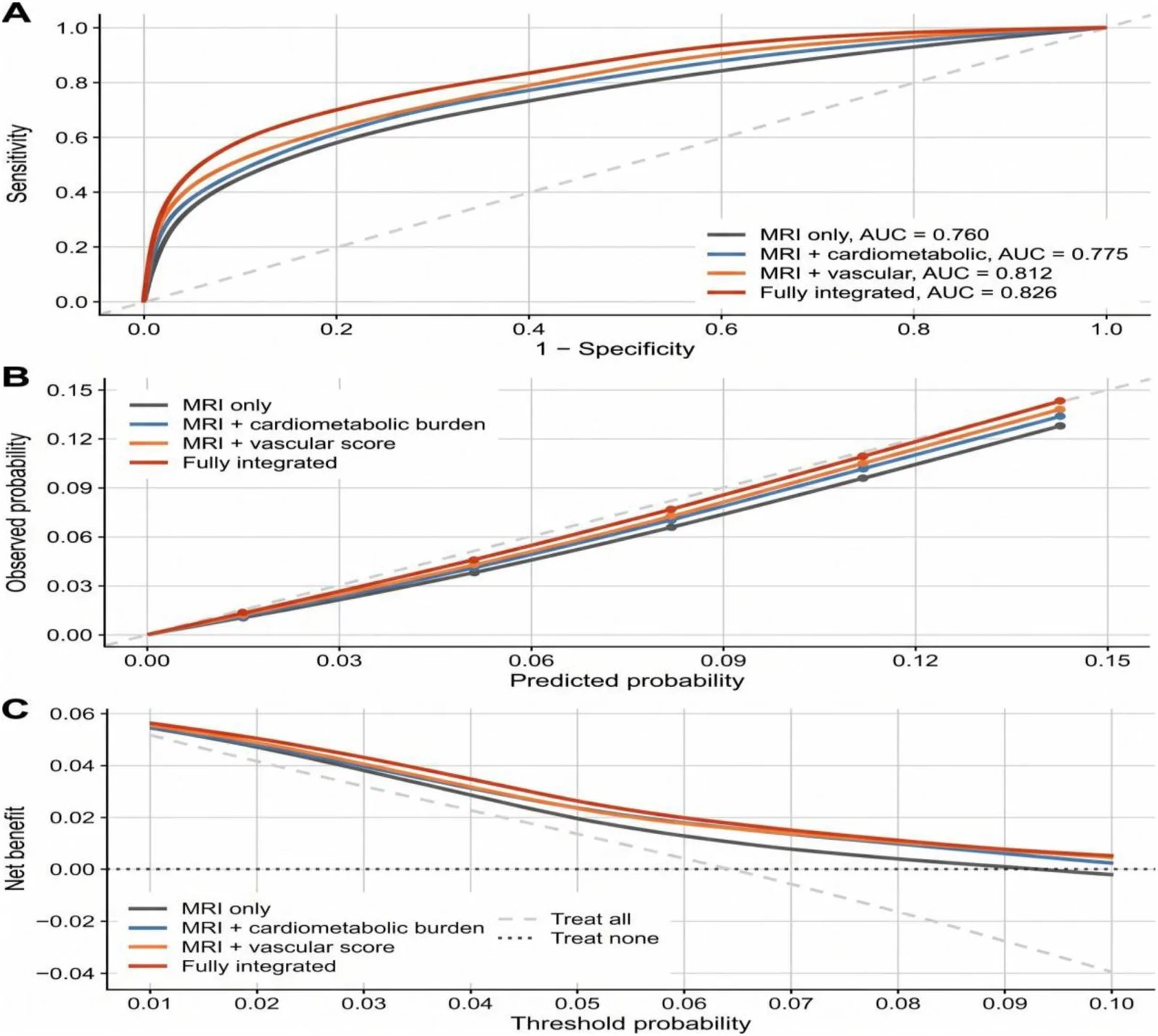
Internal model validation: **(A)** out-of-bootstrap ROC curves; **(B)** calibration curves; and **(C)** decision curves across threshold probabilities of 0.01–0.10.

### 3.4 Locked BI-RADS re-stratification

Application of the two locked thresholds changed the management assignment of 97 of 420 lesions (23.1%). Among the 260 initial BI-RADS 4A lesions, 91 (35.0%) had an aggregated out-of-bootstrap malignancy probability below 0.020 and were reassigned to 6-month MRI follow-up. These lesions comprised 90 benign lesions and 1 malignancy. The remaining 169 BI-RADS 4A lesions retained the biopsy recommendation, including 146 benign lesions and 23 of the 24 malignancies.

The locked downgrade rule would therefore have avoided immediate biopsy recommendations for 90 of 236 benign BI-RADS 4A lesions, corresponding to a benign-biopsy reduction of 38.1% (95% CI 32.2%–44.5%). One of the 24 malignant BI-RADS 4A lesions was downgraded, giving a malignant downgrade rate of 4.2% (95% CI 0.7%–20.2%). The downgraded malignant lesion nevertheless underwent tissue diagnosis under the original BI-RADS 4A pathway; the model-based reassignment was retrospective and did not alter actual clinical management.

Among the 160 initial BI-RADS 3 lesions, 6 had a predicted malignancy probability of at least 0.050 and were flagged for intensified assessment. The flagged group contained 2 malignant and 4 benign lesions. Of the 154 lesions remaining under routine short-interval surveillance, 153 were benign and 1 was malignant. The alert rule therefore identified 2 of the 3 malignant BI-RADS 3 lesions, corresponding to a detection proportion of 66.7% (95% CI 20.8%–93.9%). Because this estimate was based on only three malignant BI-RADS 3 lesions, it should be interpreted as hypothesis-generating rather than as evidence of clinical detection performance.

Across the complete cohort, the locked integrated strategy assigned 175 lesions to biopsy or intensified assessment and 245 to imaging follow-up. Twenty-five of the 27 malignancies were test-positive, while 243 of the 393 benign lesions were test-negative. Sensitivity was 92.6% (95% CI 76.6%–97.9%), specificity was 61.8% (95% CI 56.9%–66.5%), positive predictive value was 14.3% (95% CI 9.9%–20.2%), and negative predictive value was 99.2% (95% CI 97.1%–99.8%). The complete reclassification matrix and comparison with the baseline BI-RADS strategy are presented in **Table 6**. Movement from the initial BI-RADS categories to the locked integrated assignments is shown in **Figure 3**.

**Table 6:** Clinical reclassification under the locked integrated strategy.

Initial category and integrated decision		Benign	Malignant	Total		
BI-RADS 4A downgraded to 6-month MRI follow-up		90	1	91		
BI-RADS 4A retained for biopsy		146	23	169		
BI-RADS 3 flagged for intensified assessment		4	2	6		
BI-RADS 3 retained in short-interval follow-up		153	1	154		
**Total**		**393**	**27**	**420**		

Diagnostic performance comparison						
Performance measure		Baseline BI-RADS strategy		Locked integrated strategy		
True positive		24		25		
False negative		3		2		
True negative		157		243		
False positive		236		150		
Sensitivity		88.9% (71.9%–96.1%)		92.6% (76.6%–97.9%)		
Specificity		39.9% (35.2%–44.9%)		61.8% (56.9%–66.5%)		
Positive predictive value		9.2% (6.3%–13.4%)		14.3% (9.9%–20.2%)		
Negative predictive value		98.1% (94.6%–99.4%)		99.2% (97.1%–99.8%)		
Benign BI-RADS 4A biopsy recommendations avoided		0/236 (0.0%)		90/236 (38.1%; 32.2%–44.5%)		
Malignant BI-RADS 4A lesions downgraded		0/24 (0.0%)		1/24 (4.2%; 0.7%–20.2%)		
Malignant BI-RADS 3 lesions flagged		0/3 (0.0%)		2/3 (66.7%; 20.8%–93.9%)		

Values in parentheses are Wilson 95% confidence intervals. Intensified assessment comprised targeted ultrasound, focused radiologic review, earlier MRI surveillance, or biopsy when a concordant suspicious finding was identified; it did not constitute an automatic biopsy recommendation. BI-RADS, Breast Imaging Reporting and Data System; MRI, magnetic resonance imaging. Bold values indicate the total numbers of benign lesions, malignant lesions, and all lesions, respectively.

**Figure 3:**
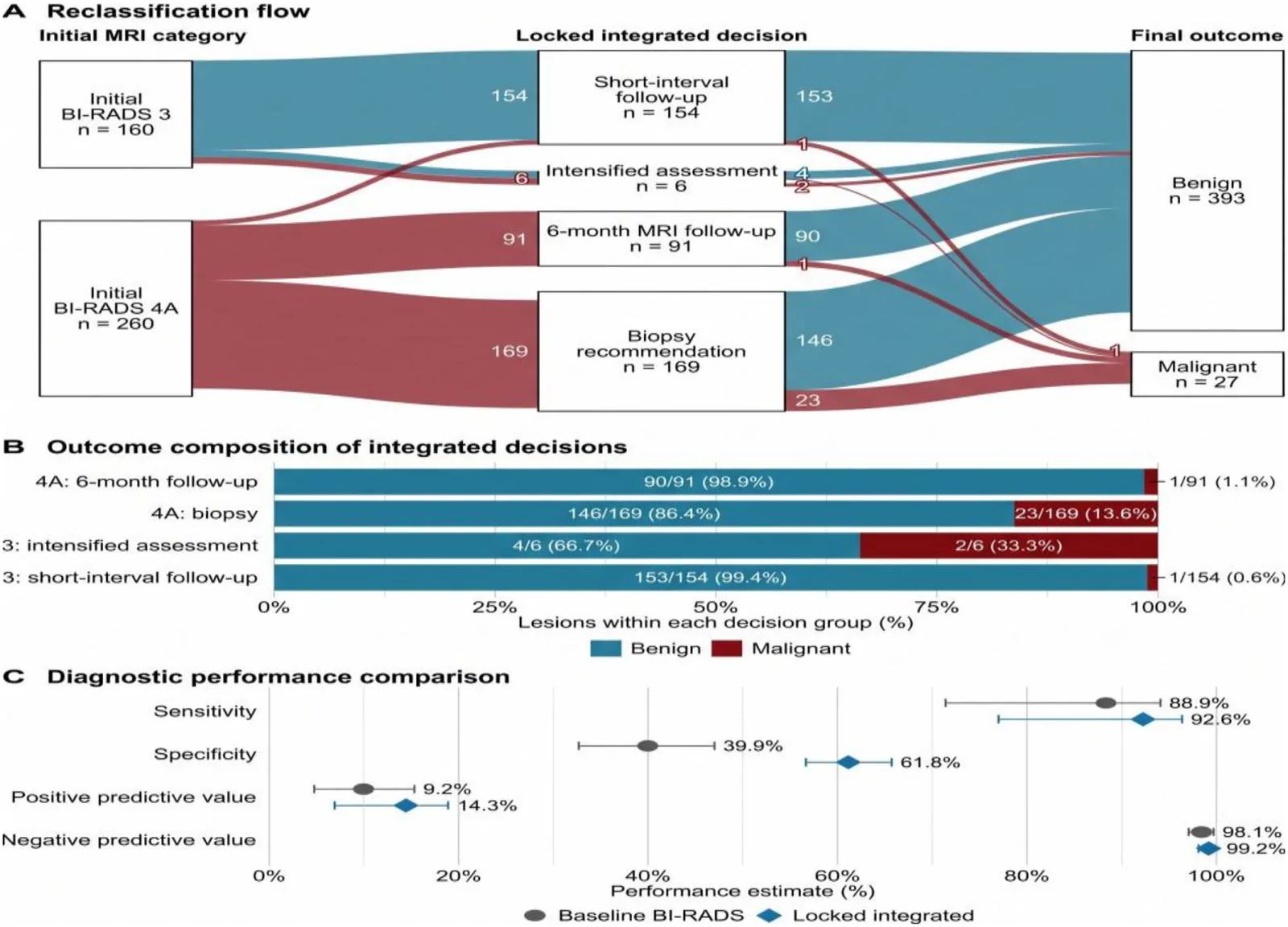
Movement of initial BI-RADS 3 and 4A lesions to follow-up, intensified assessment, or biopsy under the locked integrated strategy. **(A)** Reclassification flow from initial BI-RADS categories through integrated decisions to final benign or malignant outcomes. **(B)** Benign and malignant lesion counts and proportions within each integrated decision group. **(C)** Comparison of sensitivity, specificity, positive predictive value, and negative predictive value between the baseline BI-RADS strategy and the locked integrated strategy.

### 3.5 Decision-curve and sensitivity analysis

Across threshold probabilities from 0.01 to 0.10, the fully integrated model provided greater net benefit than the MRI-only model. At threshold probabilities of 0.02, 0.05, 0.08, and 0.10, the net benefits of the fully integrated model were 0.051, 0.026, 0.012, and 0.005, respectively, compared with 0.047, 0.018, 0.004, and −0.002 for the MRI-only model. The corresponding decision curves are presented in **Figure 2C**.

Discrimination remained similar after the prespecified image-quality exclusions. Excluding 20 examinations with grade-3 motion artifact retained 400 lesions and all 27 malignancies and produced an optimism-corrected integrated-model AUC of 0.828. Excluding 19 examinations with limited segmentation quality retained 401 lesions and 26 malignancies and produced an AUC of 0.830. Restriction to the 378 examinations with evaluable DWI, including 24 malignancies, yielded an AUC of 0.831. Each of these analyses retained one malignant BI-RADS 4A downgrade under the locked rule.

The histopathology-only analysis restricted the original locked aggregated out-of-bootstrap predictions to the 291 histopathologically verified lesions, including all 27 malignancies, without refitting the model. The AUC in this restricted evaluation was 0.811. The locked rule yielded 25 true positives, 150 false positives, 114 true negatives, and 2 false negatives; sensitivity was 92.6%, specificity 43.2%, positive predictive value 14.3%, and negative predictive value 98.3% (95% CI 93.9%-99.5%). The BI-RADS 4A downgrade count remained 91 (90 benign and 1 malignant), and the BI-RADS 3 alert count remained 6 (4 benign and 2 malignant). Adding scanner vendor, field strength, temporal resolution, and protocol variant to the integrated model resulted in an AUC of 0.824, while additional adjustment for age, menopausal status, ACR density, hormonal exposure, and family history resulted in an AUC of 0.822. Adding moderate or marked BPE as a separate predictor produced an AUC of 0.827. Using HbA1c-based glucose classification among 409 participants with available HbA1c produced an AUC of 0.823, and repeating the primary analysis after multiple imputation with 50 datasets produced a pooled AUC of 0.825. The complete sensitivity estimates are summarized in **Table 7**.

**Table 7:** Sensitivity, robustness, and threshold analyses.

Analysis	Analytic n	Malignant n	Internally validated integrated-model AUC	Additional result
Primary cohort	420	27	0.826	90 benign and 1 malignant 4A lesion downgraded
Excluding grade-3 motion artifact	400	27	0.828	One malignant 4A downgrade retained
Excluding limited segmentation quality	401	26	0.830	One malignant 4A downgrade retained
DWI-complete examinations	378	24	0.831	One malignant 4A downgrade retained
Histopathology-only cohort	291	27	0.811	Removed follow-up-only reference outcomes; locked OOB rule: TP 25, FP 150, TN 114, FN 2; NPV 98.3% (95% CI 93.9%-99.5%); 4A downgrade and BI-RADS 3 alert counts unchanged
Scanner/protocol-adjusted model	420	27	0.824	Adjusted for vendor, field strength, temporal resolution, and protocol variant
Clinical-confounder-adjusted model	420	27	0.822	Adjusted for age, menopause, ACR density, hormonal exposure, and family history
BPE-added model	420	27	0.827	Moderate or marked BPE entered separately
HbA1c-based glucose analysis	409	26	0.823	Glucose component recoded using available HbA1c
Multiple imputation, 50 datasets	420	27	0.825	Pooled across imputed datasets

BI-RADS 4A downgrade-threshold analysis				
Risk threshold	4A lesions downgraded	Benign downgraded	Malignant downgraded	Benign 4A biopsy reduction
0.010	55	55	0	23.3%
0.015	73	72	1	30.5%
0.020, locked	91	90	1	38.1%
0.025	109	107	2	45.3%

BI-RADS 3 intensified-assessment threshold analysis				
Risk threshold	BI-RADS 3 lesions flagged	Benign flagged	Malignant flagged	Malignant BI-RADS 3 lesions identified
0.030	12	9	3	3/3 (100.0%)
0.050, locked	6	4	2	2/3 (66.7%)
0.075	3	2	1	1/3 (33.3%)

AUC estimates were obtained by repeating the complete preprocessing, penalty selection, and model-fitting procedure within the bootstrap validation. Threshold analysis used aggregated out-of-bootstrap probabilities and did not replace the prespecified thresholds of 0.020 for BI-RADS 4A downgrade and 0.050 for BI-RADS 3 intensified assessment. ACR, American College of Radiology; AUC, area under the receiver operating characteristic curve; BI-RADS, Breast Imaging Reporting and Data System; BPE, background parenchymal enhancement; DWI, diffusion-weighted imaging.

Threshold analysis showed the expected trade-off between avoided benign biopsy recommendations and malignant downgrades. Lowering the BI-RADS 4A threshold to 0.010 would have downgraded 55 benign lesions without downgrading a malignancy. At 0.015, 72 benign lesions and 1 malignancy would have been downgraded. The locked threshold of 0.020 downgraded 90 benign lesions and 1 malignancy, whereas increasing the threshold to 0.025 would have downgraded 107 benign lesions and 2 malignancies. For initial BI-RADS 3 lesions, alert thresholds of 0.030, 0.050, and 0.075 flagged 12, 6, and 3 lesions, respectively. These groups contained 3, 2, and 1 of the 3 malignant BI-RADS 3 lesions.

## 4 Discussion

This study examined whether cardiometabolic burden and an MRI-derived vascular remodeling score could refine management within the narrow BI-RADS 3/4A interval. The malignancy rate was 1.9% among initial BI-RADS 3 lesions, close to the pooled rate of approximately 1.6% reported for MRI BI-RADS 3 findings, for which malignancies may be detected throughout 24 months of surveillance (Spick et al., 2018). The 9.2% rate among BI-RADS 4A lesions confirmed that this adjacent category contains a much larger benign majority but cannot be managed routinely by surveillance. After correction for optimism, AUC increased from 0.760 for the MRI-only model to 0.775 with cardiometabolic burden, 0.812 with the vascular remodeling score, and 0.826 with both additions. The fully integrated model exceeded the MRI-only model by 0.066 (95% CI 0.019–0.113; p = 0.006), with a calibration intercept of −0.08 and slope of 0.94. The pattern across the four models showed a larger observed increment after adding the vascular score, whereas the cardiometabolic increment was smaller and imprecisely estimated.

ADC remained relevant within the MRI predictor block. Malignant lesions had lower ADC values, consistent with prior evidence that quantitative DWI can increase specificity after DCE-MRI (Dijkstra et al., 2016), and multicenter data have shown that DWI can reduce unnecessary MRI-prompted biopsies (Rahbar et al., 2019). A separate multicenter study showed that a predefined ADC cutoff could downgrade a proportion of BI-RADS 4 lesions while preserving high sensitivity (Clauser et al., 2021). A later large multicenter database also supported development of an ADC category system (Bickel et al., 2023). Its use as an isolated cutoff remains difficult because ADC distributions overlap and depend on acquisition, lesion segmentation, high-b-value selection, and ROI placement. The present model therefore retained ADC as a continuous predictor and included DWI availability explicitly, allowing the 42 DCE-only examinations to remain in the primary analysis. Discrimination was similar in the DWI-complete analysis, with an optimism-corrected AUC of 0.831. The threshold analysis also demonstrated why apparent gains in biopsy reduction require caution. Increasing the BI-RADS 4A downgrade threshold from 0.020 to 0.025 increased the number of benign downgrades from 90 to 107 but increased malignant downgrades from one to two. The locked 0.020 threshold represented the more conservative operating point in this cohort.

The vascular findings extended beyond enhancement magnitude. Adjacent vessel signs and greater vessel tortuosity were more common among malignant lesions, and the microvascular remodeling score was higher, although the distributions of perilesional vessel density still overlapped. Previous histologically verified MRI research has associated the adjacent vessel sign with malignancy but found insufficient sensitivity for independent diagnostic use (Fischer et al., 2006). Individual radiomics studies of BI-RADS 4 lesions have reported encouraging discrimination, while meta-analytic evidence has also shown substantial heterogeneity across acquisition, segmentation, feature construction, and validation (Lyu et al., 2023; Lin et al., 2024). In the present study, individual vascular components were combined in a prespecified score and were not entered simultaneously with the derived phenotype, limiting predictor redundancy. The MRI-plus-vascular model reached an optimism-corrected AUC of 0.812, whereas adding cardiometabolic burden increased it to 0.826; the resulting difference of 0.014 remained uncertain. These findings suggest that the larger observed incremental gain was associated with the vascular score, but they do not establish that vascular information is definitively superior because the fully integrated-versus-vascular-only difference was small and its confidence interval crossed zero. An MRI-derived remodeling pattern cannot be assumed to represent histologic angiogenesis without direct tissue correlation.

Cardiometabolic burden showed a more limited relationship with lesion outcome. Metabolic syndrome was more frequent among malignant lesions, but BMI, waist circumference, metabolic-component count, and the three-level cardiometabolic risk distribution showed substantial overlap. Adding cardiometabolic component count to the MRI-only model increased the optimism-corrected AUC by only 0.015, and the confidence interval included no improvement. This result does not support cardiometabolic status as an independent lesion-level cancer marker. BPE is associated with several breast cancer risk factors (Brooks et al., 2022), and obesity and metabolic dysfunction correlate with greater background parenchymal enhancement, particularly in premenopausal women (Brown et al., 2023). Moderate or marked BPE may affect MRI sensitivity and specificity (Bechyna and Baltzer, 2025). BPE itself was not associated with malignancy in this cohort, and adding BPE separately to the integrated model changed the AUC only from 0.826 to 0.827. Cardiometabolic variables therefore appear to provide modest contextual adjustment to lesion-level MRI findings rather than a direct basis for biopsy or surveillance. The component-count formulation was deliberately parsimonious, but dichotomization and equal weighting can discard information contained in the underlying continuous measurements. With only 27 malignancies, the present data cannot distinguish confidently between a limitation of this score representation and a genuinely small cardiometabolic contribution.

The clinical reclassification results make the operating trade-off explicit. The locked integrated rule reassigned 90 of 236 benign BI-RADS 4A lesions to 6-month follow-up, reducing benign immediate biopsy recommendations by 38.1% (95% CI 32.2%–44.5%). One of the 24 malignant BI-RADS 4A lesions was also downgraded, corresponding to 4.2%, with a wide 95% confidence interval of 0.7%–20.2%. Among initial BI-RADS 3 lesions, intensified assessment identified two of the three malignancies while also flagging four benign lesions. Across the cohort, sensitivity was 92.6%, specificity was 61.8%, positive predictive value was 14.3%, and negative predictive value was 99.2%. The high negative predictive value partly reflects the low prevalence of malignancy and does not negate the clinical importance of the malignant downgrade. Decision-curve analysis showed higher net benefit for the integrated model across the evaluated threshold range, but these calculations were based on internally validated predictions. The model currently supports structured risk modification within BI-RADS management, not replacement of radiologist assessment, tissue diagnosis, or established surveillance. Because the reassignment was retrospective, the downgraded malignant BI-RADS 4A lesion actually underwent tissue diagnosis under the original clinical pathway. Given the denominator of only three malignant BI-RADS 3 lesions, the 2-of-3 alert result is hypothesis-generating.

The study has several limitations. Its retrospective single-center design leaves potential selection, spectrum, and verification effects. All BI-RADS 4A lesions underwent tissue diagnosis, whereas most benign BI-RADS 3 outcomes were assigned after imaging stability, creating different reference-standard pathways between categories. Twenty-four months of stability is accepted for BI-RADS 3 surveillance but may not exclude every slow-growing lesion. Cardiometabolic measurements obtained within 30 days before or after MRI did not represent simultaneous physiological assessment, and residual confounding may remain despite technical and clinical sensitivity analyses. Scanner heterogeneity, automated vessel extraction, and perilesional segmentation may also affect transportability. Only 27 malignant outcomes were available. Restricting the fully integrated model to eight effective coefficients, applying ridge penalization, repeating the complete modeling process within 2,000 bootstrap resamples reduced overfitting risk but could not remove the uncertainty associated with the small event count. Aggregated out-of-bootstrap predictions provide internal validation rather than evidence of performance at another institution. The calibration slope of 0.94 for the integrated model remained below 1 after optimism correction, indicating residual overfitting and suggesting that external use would likely require recalibration in addition to validation. When the original locked out-of-bootstrap predictions were restricted to the 291 histopathologically verified lesions without refitting, negative predictive value remained high at 98.3%, but specificity decreased to 43.2% after removal of follow-up-only benign cases. This analysis quantifies the influence of reference-standard composition but cannot eliminate differential verification bias because biopsy was not randomly assigned among BI-RADS 3 lesions. Prospective multicenter validation should prospectively test the prespecified 0.020 and 0.050 thresholds without outcome-driven re-optimization, evaluate whether probability recalibration is required, use harmonized image processing, report calibration by scanner and clinical subgroup, and document every malignant lesion assigned to surveillance before management changes are considered.

## 5 Conclusion

Combining conventional MRI predictors with cardiometabolic burden and an MRI-derived vascular remodeling score improved internally validated re-stratification of BI-RADS 3 and 4A breast lesions. The larger observed incremental gain was associated with the vascular score, whereas the cardiometabolic increment was smaller and imprecisely estimated. At the locked thresholds, the strategy retrospectively reassigned 38.1% of benign BI-RADS 4A lesions away from immediate biopsy and flagged 2 of 3 malignant BI-RADS 3 lesions; the latter finding is hypothesis-generating because only three malignant BI-RADS 3 lesions were present. One malignant BI-RADS 4A lesion would also have been reassigned to follow-up, and the wide uncertainty around this safety estimate, together with residual calibration overfitting, precludes clinical implementation. Independent prospective multicenter validation and likely recalibration are required before the model can be considered for clinical use.

## Data Availability

The original contributions presented in the study are included in the article/**Supplementary Material**, further inquiries can be directed to the corresponding author.
