# Supplementary material for: Re-stratification of BI-RADS 3/4A breast lesions based on dynamic contrast-enhanced MRI: integrating cardiometabolic risk and breast microvascular remodeling phenotypes to improve diagnostic accuracy and reduce unnecessary biopsies
Source: Front Physiol. 2026 Oct 28;17:1984062. doi: 10.3389/fphys.2026.1984062 (PMC13751330; doi:10.3389/fphys.2026.1984062)
Supplement: Supplementary file 1 [file SupplementaryFile1.docx]

Supplementary Material

# 1 Supplementary Figures and Tables

## 1.1 Supplementary Figure

**Supplementary Figure S1.** Segmentation and vascular-feature workflow. (**A**) Index lesion. (**B**) Three-dimensional lesion mask. (**C**) Five-millimeter perilesional shell. (**D**) Vesselness map and centerlines. (**E**) Vascular measurements. (**F**) Repeat-segmentation adjudication.

## 1.2 Supplementary Table

**Supplementary Table S1.** Variable Dictionary, Missing-Data Rules, and Sensitivity Analyses

| **Variable group** | **Variables** | **Format** | **Primary rule** |
| --- | --- | --- | --- |
| Record linkage | Study ID, lesion ID, examination date, laterality | Text, date, categorical | Required |
| Reference outcome | Reference type, date, pathology, final outcome | Categorical, date, binary | Required |
| Participant profile | Age, menopause, parity, family history, previous biopsy, hormonal exposure | Continuous or categorical | Sensitivity adjustment |
| Cardiometabolic data | BMI, waist, blood pressure, glucose, HbA1c, triglycerides, HDL, LDL, treatment | Continuous or binary | Closest value within ±30 d |
| Cardiometabolic burden | Abnormal-component count | Continuous, 0–5 | Sole primary cardiometabolic predictor |
| Scanner variables | Vendor, field strength, temporal resolution | Categorical or continuous | Technical sensitivity adjustment |
| Reader variables | Reader 1, reader 2, adjudicated BI-RADS | Categorical | Agreement and model predictor |
| Breast composition | ACR density, fibroglandular tissue amount, BPE | Categorical | Description and sensitivity analysis |
| Primary MRI variables | Adjudicated BI-RADS, diameter, morphology indicator, iAUC60, ADC, DWI availability | Continuous or binary | MRI predictor block |
| Exploratory DCE variables | Initial enhancement, delayed slope, SER, $\text{(}\text{K}^{\text{trans}}\text{),(}\text{k}_{\text{ep}}\text{),(}\text{v}_{\text{e}}\text{),(}\text{v}_{\text{p}}\text{)}$ | Continuous | Descriptive and sensitivity analysis |
| Vascular components | Adjacent vessel, asymmetry, density, rim enhancement, edema, tortuosity | Binary or continuous | Used only to derive vascular score |
| Vascular score | MRI-derived vascular remodeling score | Continuous, 0–10 | Sole primary vascular predictor |
| Descriptive phenotype | Quiescent, adaptive, remodeled | Categorical | Reporting only |
| Quality variables | Motion grade, segmentation quality, DWI availability | Categorical | Sensitivity exclusions |
| Missing values | Retained continuous and binary variables | Variable-specific | Bootstrap median or mode |
| Model outputs | Four predicted risks and reclassification assignment | Continuous or categorical | Derived during validation |

Sensitivity-analysis rules

| **Analysis** | **Rule** | **Purpose** |
| --- | --- | --- |
| Motion-artifact analysis | Exclude grade-3 examinations | Image-quality robustness |
| Segmentation analysis | Exclude limited segmentation | ROI robustness |
| DWI-complete analysis | Exclude examinations without evaluable ADC | Diffusion dependence |
| Histology-only analysis | Restrict to 291 histopathologically verified lesions; original locked aggregated out-of-bootstrap predictions retained; no model refitting | Differential-verification assessment |
| Technical adjustment | Add vendor, field strength, and temporal resolution | Scanner robustness |
| Clinical adjustment | Add age, menopause, ACR density, hormonal exposure, and family history | Clinical confounding |
| BPE-added analysis | Add BPE outside the vascular score | Parenchymal contribution |
| HbA1c analysis | Count HbA1c ≥5.7% as elevated glucose | Glucose-definition robustness |
| Multiple-imputation analysis | Fifty chained-equation datasets | Imputation robustness |
| 4A threshold analysis | 0.010, 0.015, 0.020, and 0.025 | Downgrade trade-off |
| BI-RADS 3 threshold analysis | 0.030, 0.050, and 0.075 | Alert trade-off |

Note. ACR, American College of Radiology; ADC, apparent diffusion coefficient; BI-RADS, Breast Imaging Reporting and Data System; BMI, body mass index; BPE, background parenchymal enhancement; DCE, dynamic contrast-enhanced; DWI, diffusion-weighted imaging; HDL, high-density lipoprotein; iAUC60, initial area under the enhancement curve during the first 60 s; LDL, low-density lipoprotein; MRI, magnetic resonance imaging; ROI, region of interest; SER, signal enhancement ratio.
